# Reciprocal Regulation between Primary Cilia and mTORC1

**DOI:** 10.3390/genes11060711

**Published:** 2020-06-26

**Authors:** Yandong Lai, Yu Jiang

**Affiliations:** Department of Pharmacology and Chemical Biology, University of Pittsburgh School of Medicine, Pittsburgh, PA 15261, USA; yandong.lai@pitt.edu

**Keywords:** primary cilia, mTOR, mTORC1, autophagy, ciliogenesis, Tsc2, polycystin-1, LKB1, AMPK

## Abstract

In quiescent cells, primary cilia function as a mechanosensor that converts mechanic signals into chemical activities. This unique organelle plays a critical role in restricting mechanistic target of rapamycin complex 1 (mTORC1) signaling, which is essential for quiescent cells to maintain their quiescence. Multiple mechanisms have been identified that mediate the inhibitory effect of primary cilia on mTORC1 signaling. These mechanisms depend on several tumor suppressor proteins localized within the ciliary compartment, including liver kinase B1 (LKB1), AMP-activated protein kinase (AMPK), polycystin-1, and polycystin-2. Conversely, changes in mTORC1 activity are able to affect ciliogenesis and stability indirectly through autophagy. In this review, we summarize recent advances in our understanding of the reciprocal regulation of mTORC1 and primary cilia.

## 1. Introduction

Quiescence is a state of reversible cell cycle arrest and a stage when a cell acquires its specialty for sustaining tissue and organ functions. To enter and maintain quiescence, a cell needs to adapt a metabolic state that differs from that when it is in the cell cycle. As a master regulator of cell metabolism, the mechanistic target of rapamycin complex 1 (mTORC1) plays an important role in the metabolic reprogramming. In comparison with cycling cells, quiescent cells have a reduced mTORC1 activity, which is essential for maintaining the quiescent state. Many mechanisms contribute to the downregulation of mTORC1 in quiescent cells, including diminished growth factor signaling caused by contact inhibition [1,2]. However, an increasing body of evidence suggests that primary cilia, a unique membranous protrusion at the apical surface of quiescent cells, also play an important role in restricting mTORC1 signaling activity. Primary cilia functions as a cellular antenna to sense extracellular chemical and mechanic signals. It converts mechanic signals into chemical activities for mTORC1 inhibition. This primary cilium-dependent downregulation of mTORC1 appears to be important for maintaining cell quiescence, as the abnormal activation of mTORC1 is often associated with disease conditions caused by defects in primary cilia [3,4]. On the other hand, changes in mTORC1 activity also affect the ciliogenesis and stability of primary cilia. The mutual regulation of primary cilia and mTORC1 is likely a key attribute for controlling quiescence and cell cycle entry. In this review, we summarize the molecular basis underlying the mutual regulation.

## 2. The Primary Cilium as a Signaling Hub

**Figure 1:**
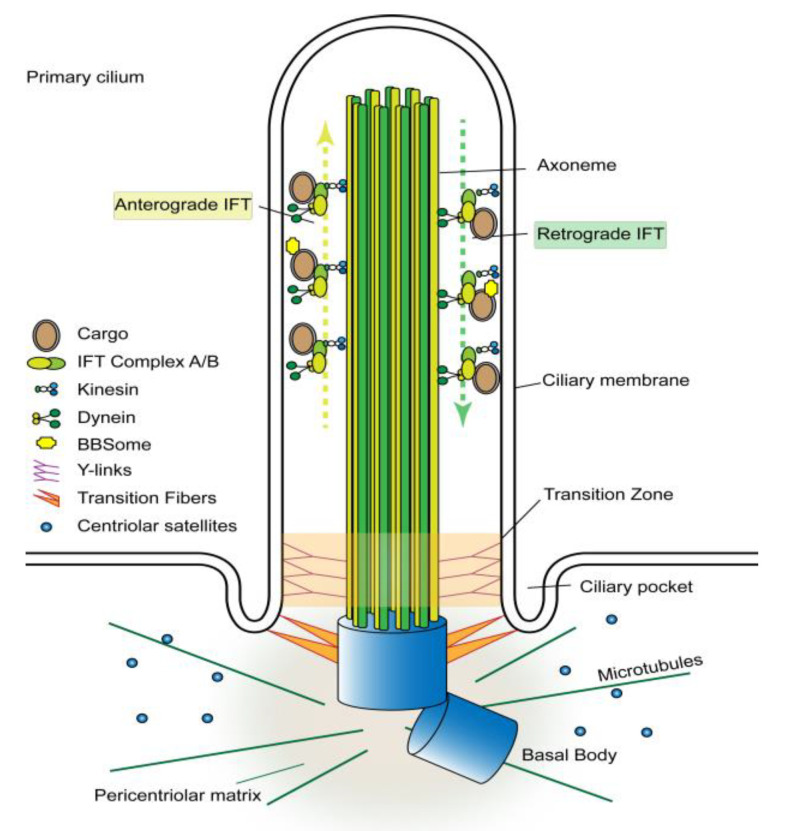
Structure of the primary cilium. The primary cilium is a solitary cell surface protrusion composed of a microtubule-based core (axoneme) ensheathed by an extension of the plasma membrane. The axoneme contains nine pairs microtubules emanating from the basal body located at the base of the cilium. The proximal ends of axonemal microtubules are tethered to the ciliary membrane by Y-links, which form the transition zone. The ciliary compartment is separated from the cytoplasm by transition fibers connecting the basal body microtubules to the ciliary membrane. The basal body also serves as a microtubule organization center that attracts pericentriolar proteins and microtubules. The transition fibers are the docking site of intraflagellar transport (IFT) particles (IFT-A/B), which carry proteins into the ciliary compartment. The loading of protein cargos to the IFT particles is mediated by BBSome. Anterograde IFT moves proteins along the axonemal microtubules from the base to the tip of the cilium and is propelled by the KIF3 kinesin motor complex, whereas, retrograde IFT transports proteins from the tip to the base of the cilium and is driven by the cytoplasmic dynein motor complex.

With some exceptions, the primary cilium is assembled when a cell exits the cell cycle and enters quiescence [5]. The assembly process consists of two stages, including the formation of a microtubule organization center and generation of the axoneme [6]. The process begins with the basal body development from the centrosome, which consists of a mother and a daughter centriole surrounded by pericentriolar material [7]. The basal body migrates toward the cell surface in association with membrane vesicles, which subsequently fuse with the plasma membrane [8]. Upon docking underneath the plasma membrane, the basal body serves as a nucleating center, attracting spherical granules called centriolar satellites, which are composed of proteins essential for basal body maintenance and ciliogenesis [9]. Bardet–Biedl Syndrome 4 (BBS4), a scaffolding protein, is then recruited to the basal body to initiate the formation of BBSome, a complex of seven Bardet–Biedl syndrome proteins [10,11]. Upon the formation of the microtubule organization center underneath the plasma membrane, nine microtubule doublets emanate from the basal body to form the axoneme. A lipid bilayer extending from the plasma membrane encases the axoneme and creates the ciliary compartment, which is separated from the cytoplasm by a transition zone at the base of the cilium [12,13] (Figure 1). The BBSome plays a specific role in protrusion of the axonemal microtubules from the basal body by acting as an adaptor for binding of protein cargos to intraflagellar transport [14,15,16]. Further assembly of this nascent cilium requires intraflagellar transport (IFT) that moves tubulins and other axonemal proteins across the transition zone into the ciliary compartment [17]. The transport is carried out by the IFT particles composed of about 20 proteins organized into two sub-complexes called A (IFT-A) and B (IFT-B). The IFT particles move bi-directionally along the axonemal microtubules. The anterograde transport (from base to tip) is driven by molecular motor heterotrimeric kinesin-2, whereas the retrograde transport (from tip to base) by cytoplasmic dynein-2 [17]. Upon the establishment of the axoneme and the ciliary compartment, the accumulation of membrane receptors and soluble signaling proteins within the ciliary compartment enable the cilium to function as a signaling hub. The accumulation is a selective process; some proteins are highly enriched while others are excluded [18]. Membrane proteins are imported into the ciliary compartment mainly by the intraflagellar transport [17,19]. How soluble proteins, particularly those of signal transduction pathways, enter and accumulate in the cilium is less clear. It has been suggested that soluble proteins can cross the transition zone either by tagging along with membrane proteins or through simple diffusion [5,20]. In the latter case, the transition zone functions as a size-exclusion permeability barrier [21,22,23].

As a ubiquitous sensory organelle, primary cilia can be found on almost all mammalian cells and play a crucial role in the cellular response to the chemical, mechanical and light stimuli [24,25]. To a certain extent, primary cilia act in a way similar to lipid rafts in that they offer a unique microdomain for regulating membrane distribution of receptors and signaling molecules. The physical protrusion of cilia into the extracellular space also provides a way for sensing mechanical signals and physical interactions with surrounding cells. The active intraflagellar transport and the limited ciliary space of cilia create an ideal environment for concentrating signaling molecules, organizing signaling cascades, and facilitating crosstalks between different pathways. Primary cilia harbor many signaling proteins that are components of various signaling pathways, including Sonic Hedgehog (Shh), Wnt, Notch, G-protein-coupled receptors (GPCR), Platelet-derived growth factor (PDGF), Hippo and mTORC1 [26,27]. Among the pathways, the Shh pathway is the major cilium-dependent pathway with its receptors and key components residing within the cilium. Other pathways, including the mTORC1 pathway, operate through both cilium-dependent and independent mechanisms.

## 3. The mTORC1 Pathway

mTOR, the mechanistic target of rapamycin, is a protein kinase that exists in two complexes termed as mTOR complex 1 (mTORC1) and 2 (mTORC2). Each complex comprises different regulatory components and performs distinct functions [28]. mTORC1, the rapamycin-sensitive complex, is composed of mTOR as the catalytic subunit, mLST8 as a structural component, and Raptor as the regulatory subunit that defines the substrate specificity of the complex [29]. mTORC2 shares mTOR and mLST8 with mTORC1 but also contains two distinct subunits, mSIN1 and Rictor, of which Rictor is the regulatory subunit dictating the specificity of the complex [30,31,32]. In addition, mTOR has been found to associate with other cellular proteins independent of mTORC1 and mTORC2 [33,34].

mTORC1 acts as a master regulator of cell metabolism by integrating signals of various origins, including growth factors, energy, and nutrients. Several tumor suppressors and oncoproteins lie upstream and convey growth factor and energy signals to mTORC1. A key regulatory factor that funnels the signals to mTORC1 is the tumor suppressor complex of Tsc1 and Tsc2. The complex inhibits mTORC1 activity by functioning as the GTPase activating protein (GAP) for small GTPase Rheb, the proximal activator of mTORC1. Under the energy-depletion condition, an increased ratio of AMP/ATP acts synergistically with the liver kinase B1 (LKB1), leading to the activation of the AMP-activated protein kinase (AMPK). The activated AMPK in turn phosphorylates Tsc2 and increases its GAP activity for inhibiting Rheb, resulting in the downregulation of mTORC1 [35]. In addition, AMPK can also downregulate mTORC1 activity by blocking the phospholipase-D-mediated production of phosphatidic acid, a second messenger that activates mTORC1 [36]. Upon growth factor stimulation, receptor tyrosine kinases activate the phosphatidylinositol 3-kinase (PI3K)-Akt pathway to promote phosphorylation of Tsc2 by Akt. The Akt-dependent phosphorylation inhibits the GAP activity of the Tsc1/Tsc2 complex, resulting in an increase in mTORC1 activity [37]. The activity of mTORC1 is also regulated indirectly by mTORC2, which phosphorylates Akt and increases its activity [38].

mTORC1 plays a pivotal role in intracellular protein turnover and homeostasis through regulating protein synthesis and autophagy. The activation of mTORC1 promotes mRNA translation by directly phosphorylating several factors involved in translation initiation, including S6 kinases and eIF4E binding proteins (4E-BPs) [39]. mTORC1 controls autophagy through the ULK1/ATG13/FIP200 complex that is essential for autophagy initiation. When mTORC1 is active, it phosphorylates both ULK1 and ATG13. The phosphorylation inhibits ULK1 activity and reduces its association with ATG13, hence preventing autophagy initiation [40].

## 4. Regulation of mTORC1 by Flow Stress

Cells with defective primary cilia have a larger cell size in comparison with their counterparts with normal cilia. The size enlargement is caused by an abnormal activation of mTORC1 [41]. In ciliated renal epithelial cells, the mTORC1 activity is restricted by fluidic flow that deflects the cilia. When cilium formation is blocked, mTORC1 activity increases and becomes unresponsive to flow stress. The flow-induced and cilium-dependent downregulation of mTORC1 requires LKB1, which localizes at the ciliary compartment and the basal body. The ciliary level of LKB1 is low in the absence of flow stress but increases in response to flow stress. The accumulation of LKB1 within the cilium activates AMPK localized at the basal body [41], which in turn phosphorylates Tsc2, leading to the inhibition of mTORC1 [42].

**Figure 2:**
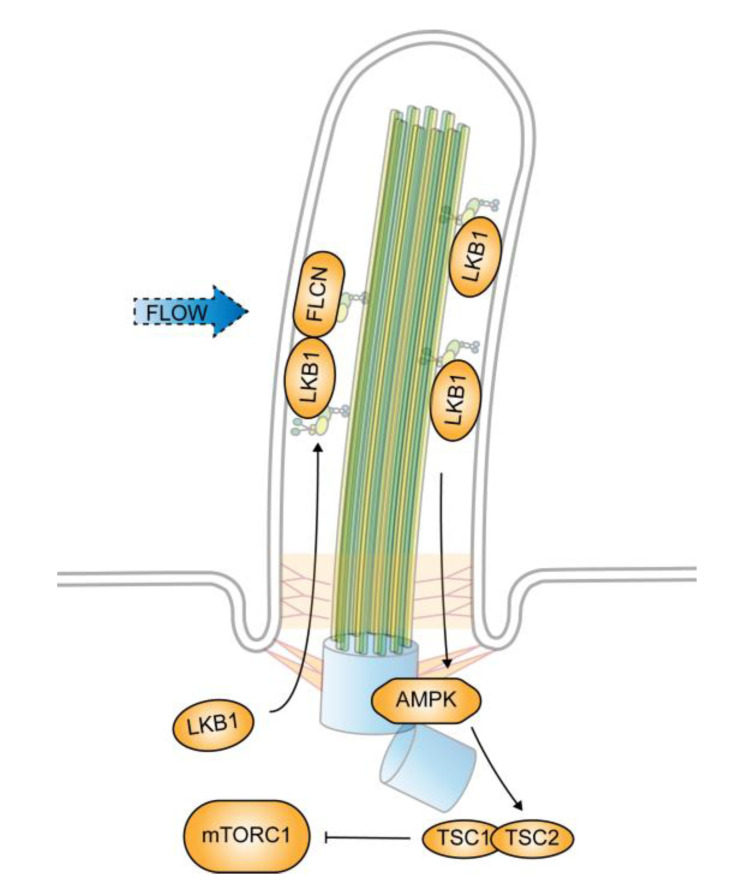
Cilium-dependent regulation of mechanistic target of rapamycin complex 1 (mTORC1) by flow stress. mTORC1 activity decreases in response to flow stress that deflects primary cilia. The decrease is caused by FLCN-mediated ciliary accumulation of liver kinase B1 (LKB1). The accumulated LKB1 activates AMP-activated protein kinase (AMPK) localized at the basal body, which in turn phosphorylates Tsc2, leading to mTORC1 downregulation.

The flow-induced accumulation of LKB1 in primary cilia is mediated by tumor suppressor folliculin (FLCN), the product of the BHD gene [42]. BHD is the causative gene for the Birt-Hogg-Dubé syndrome in humans, which is manifested by benign tumors and cystic growths in the kidneys, lungs, and skin [43]. In tumors from BHD patients and animal models, mTORC1 is hyperactivated, which is believed to be a major attribute to the pathogenesis of BHD [44,45]. FLCN resides in cilia among many other places [46]. It enters cilia through a direct binding with kinesin-2, the motor protein complex for anterograde transport in cilia, and is likely to be a cargo of kinesin-2 [42]. In the absence of FLCN, LKB1 fails to accumulate in cilia, and, consequently, AMPK remains inactive under flow stress. The mechanical signals that originate from the bending of cilia are thus blocked and mTORC1 becomes unresponsive to flow [42] (Figure 2).

How FLCN controls the ciliary accumulation of LKB1 remains unclear. Although FLCN has been found to associate with LKB1 in a cilium-dependent manner, there is no evidence that FLCN is able to directly recruit LKB1 into cilia [42]. FLCN bears structural features of regulators of small GTPases and has been proposed to function as a guanine nucleotide exchange factor or a GAP [47,48,49]. It is likely that FLCN controls the accumulation of LKB1 indirectly through regulating a ciliary small GTPase.

## 5. Regulation of mTORC1 by Polycystin-1

The first evidence that primary cilia regulate mTORC1 signaling came from the study of the mechanism underlying the pathogenesis of autosomal dominant polycystic kidney disease (ADPKD) [50]. The disease is characterized by development of many fluid-filled cysts in the kidneys, which gradually replace normal renal tissue, resulting in a progressive loss of renal function [51,52]. The aberrant activation of mTORC1 has been observed in the renal cysts from ADPKD patients and animal models of PKD [53]. Many studies have shown that the inhibition of mTORC1 decreases cyst formation and reduces kidney volume in ADPKD patients and animal PKD models [54,55,56,57,58], suggesting that an abnormal mTORC1 activity plays a key role in the progress of the disease.

ADPKD is caused by inactive mutations in the PKD1 and PKD2 genes, which, respectively, encode polycystin-1 (PC-1) and polycystin-2 (PC-2) [59]. PC-1 is a large receptor-like membrane protein consisting of 4302 amino acids with a large *N*-terminal extracellular region, 11 transmembrane domains, two large intracellular loops, and a cytoplasmic C-terminal region [60,61]. The large *N*-terminal extracellular domain is believed to be a sensor that alters conformation in response to mechanical stimuli [62]. The cytoplasmic C-terminal region of PC-1 acts as a signaling transducer through interactions with various signaling proteins, including PC-2 [63,64], β-catenin [65], Tsc2 [66], a signal transducer and activator of transcription 6 (STAT6), and its coactivator P100 [67]. The C-terminal tail of this region can also be cleaved and imported into the nucleus for transcription regulation [67,68]. In renal epithelial cells, PC-1 localizes mainly at cell adhesion structures and primary cilia. The former location is related to the function of PC-1 in mediating cell-cell or cell-matrix adhesion. However, whether this function of PC-1 contributes to its role in preventing cystic growth remains unclear [69]. Localization to primary cilia, on the other hand, is essential for the anti-cystic growth activity of PC-1. It has been found that mutations in PC-1 that alter its trafficking to primary cilia nullify its suppressor function and are pathogenic in humans and animals [70]. PC-2 is a calcium-permeable nonselective cation channel [71]. It localizes to cilia and interacts with PC-1 through its C-terminal domain [72,73]. The loss of PC-2 prevents the cilium presentation of PC-1 [70,74], suggesting that primary cilia are important for PC-1/2 function in suppressing renal cystogenesis.

Multiple mechanisms have been found that mediate the effect of PC-1 on mTORC1 in a cilium-dependent manner. The most conventional one is PC-2 and a calcium-dependent manner. Bending cilia by flow stress has been found to cause calcium influx into the cytoplasm from intracellular stores, resulting in a rise in cytoplasmic calcium level [75]. This flow-induced and cilium-dependent rise in cytoplasmic calcium requires functional PC1 and PC2 localized at cilia [76]. It is believed that PC-1 functions as a mechanosensor to stimulate PC-2 dependent calcium entry into the ciliary compartment, which in turn triggers the cytoplasmic calcium response [52]. The increased cytoplasmic calcium concentration inhibits adenylyl cyclases [77,78], hence reducing intracellular cyclic adenosine monophosphate (cAMP) levels. These connections have led to the current view regarding how a loss of PC-1 is linked to an abnormally activated mTORC1. It depicts that in the absence of PC-1/PC-2 activity, the level of cytoplasmic calcium decreases, resulting in an increase in adenylyl cyclase activity and a higher level of cAMP. The elevated level of cAMP then activates protein kinase A (PKA), which in turn acts through ERK1/2 to inhibit Tsc2 and consequently increases mTORC1 activity [79].

**Figure 3:**
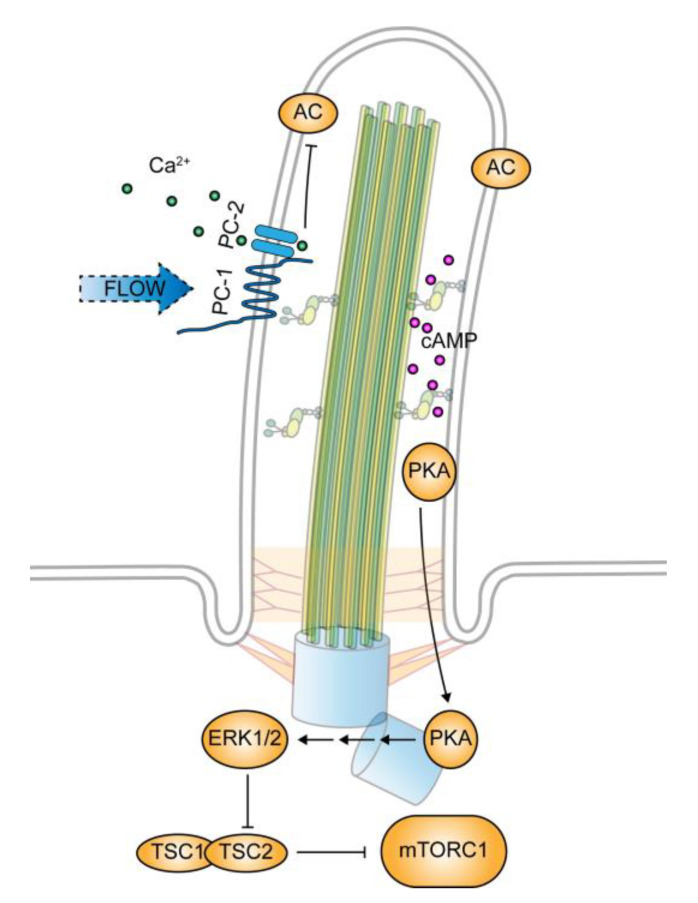
Regulation of mTORC1 by polycystin-1. Polycystin-1 (PC-1) functions as a mechanosensory protein in primary cilia. Flow stress that bends cilia activates PC-1, which in turn stimulates PC-2-mediated calcium influx into the ciliary compartment. The increased ciliary calcium level inhibits cilium-localized adenylyl cyclases (AC) and reduces the cyclic adenosine monophosphate (cAMP) level within the ciliary compartment. The cessation of flow stress or PC-1 dysfunction blocks the calcium entry and reduces the ciliary calcium level. Consequently, adenylyl cyclase activity increases and drives the ciliary cAMP level high, which activates PKA, leading to mTORC1 downregulation through the ERK1/2 and Tsc2 signaling cascade.

One caveat of this model is that an increase in ciliary calcium level may not be able to trigger the release of calcium from their storages in the cytoplasm. While the cilium is required for the increase of calcium in the cytoplasm [76], changes in ciliary calcium levels are found to have little effect on cytoplasmic calcium levels [80]. It is possible that the PC-1 to PKA signaling cascade takes place within the ciliary compartment. In support of the view, the key components of the PKA cascade are found in cilia, including adenylyl cyclases and PKA, and that the ciliary compartment has a high concentration of cAMP [18,81] (Figure 3).

The second mechanism involves STAT6 through a forward feedback signaling loop. PC-1 has been found to associate with transcription factor STAT6 and its coactivator P100 within primary cilia [67]. The association is mediated by the C-terminal cytoplasmic region of PC-1. In renal epithelial cells, upon the cessation of fluid flow that deflects cilia, the PC-1 C-terminal cytoplasmic region undergoes a proteolytic cleavage that releases the tail half. The released half, together with STAT6 and P100, translocate into the nucleus, where they stimulate the expression of IL13 receptor. The overproduced receptor then leads a higher receptor signaling through PI3K and Akt, and eventually mTORC1 activation [82].

In addition, PC-1 has been shown to interact with Tsc2 directly through its C-terminal cytoplasmic region [66]. This interaction tethers Tsc2 to the plasma membrane where it forms the complex with Tsc1 to inhibit Rheb. Phosphorylation of Tsc2 by Akt blocks the interaction and releases Tsc2 from the membrane. The dislocation of Tsc2 reduces its interaction with membrane-bound Rheb and consequently attenuates its inhibitory effect on the small GTPase. However, this mechanism does not appear to be dependent on primary cilia, as the association takes place in the absence of cilia, at least, when the PC-1 C-terminal is ectopically expressed.

The mechanisms mentioned above effectively explain how the loss of PC-1 and PC-2 function lead to the abnormal activation of mTORC1, which is commonly observed in renal cysts of PKD. However, they are largely built on correlations between a defective PC-1 with an abnormally activated mTORC1. Direct evidence showing PC-1 or PC-2 controls mTORC1 in a cilium-dependent manner under physiological conditions remains scarce. A recent study from Kuehn’s group provides the first evidence showing a direct impact of PC-1 on mTORC1 activity [83]. It was found that flow stress-induced mTORC1 downregulation required PC-1, but not PC-2. The PC-2 independent regulation of mTORC1 excludes calcium as a mediator in this mechanosensory function of PC-1. However, how PC-1 transmits the mechanical signal to mTORC1 remains unknown. This PC-2-independent mechanosensory mechanism may involve LKB1 and its target AMPK. Consistent with the view, a previous study from the same group has shown that PC-1 is able to associate with LKB1 [84].

While the cilium-localized PC-1 is able to control mTORC1 activity through multiple mechanisms, changes in mTORC1 activity can also alter the ciliary presentation of PC-1. It was found that in Tsc1-deficient mouse embryonic fibroblasts (MEFs), the level of PC-1 was reduced. The reduced expression was restored upon treatment of the deficient cells with rapamycin, indicating that mTORC1 negatively controls the expression levels of PC-1. The absence of Tsc1 also reduced the ciliary presentation of the PC-1/PC-2 complex, which was prevented by the rapamycin treatment [85]. In addition, the trafficking and cell surface presentation of PC-1 are also controlled by Tsc2 [86]. In Tsc2-deficient polycystic kidney cells, PC-1 was found to be trapped in the Golgi apparatus and unable to traffic to the plasma membrane. Although there is no direct evidence showing the effect of Tsc2 deficiency on the ciliary presentation of PC-1, given the fact that PC-1 was unable to traffic out of the Golgi apparatus, it is likely that the ciliary presentation of PC-1 is blocked in Tsc2 deficient cells. However, it remains unclear whether the effect of Tsc2 on PC-1 trafficking is mediated by mTORC1.

## 6. Regulation of Cilium Length by mTORC1

Changes in mTORC1 activity have profound effects on both ciliation frequency and cilium length. Ciliation frequency is the ratio of ciliated cells in a cell population, which is determined by the rate of ciliogenesis. The length, on the other hand, is determined by cilium growth and stability. The two processes are mechanistically independent of each other.

Both Tsc1- and Tsc2-deficient cells have been found to possess longer cilia than their proficient counterparts [87,88,89]. The activation of mTORC1 either by Rheb overexpression or by enhanced RagA, an activator of mTORC1 in nutrient signaling, also promotes longer cilia [90,91]. Conversely, the inhibition of mTORC1 by rapamycin results in shortened cilia [89]. These findings appear to suggest that mTORC1 plays a positive role in maintaining cilium stability and/or cilium growth. While the underlying mechanism for the positive effect of mTORC1 on cilium length remains unclear, it has been suggested that mTORC1 promotes cilium elongation indirectly by increasing protein synthesis [92]. However, many other studies have found opposite effects of mTORC1 on cilium length. Shortened cilia were observed in Tsc2-null cells and longer cilia were induced by rapamycin [89,93]. In addition, rapamycin appears to increase cilium length in a dose-dependent manner [93]. The reason for these discrepancies is not yet clear. However, it highlights the complicity involved in controlling cilium growth and stability. Different cells respond differently to mTORC1 downregulation owing to their distinct composition of mTORC1 related signaling components [94]. The cell content-dependent effects also suggest that mTORC1 plays an indirect role in ciliation, likely through controlling protein synthesis, turnover, or the metabolic fitness of the cells.

## 7. Regulation of Ciliogenesis by mTORC1

The formation of cilia takes place when cells exit the cell cycle upon nutrient or growth factor limitation. How ciliogenesis is coupled to nutrient and growth factor availability is currently unclear. However, findings from many different studies suggest that autophagy, a key downstream event of mTORC1, serves as a link.

Autophagy is the main process for the regulation of intracellular proteins and organelles’ turnover to achieve energetic balance in response to stress [95]. Autophagy occurs constitutively at the basal level in almost all cells but is enhanced when growth factor or nutrient signaling is diminished [96]. While it functions as a relatively non-selective cell recycling process, autophagy can promote ciliogenesis by the selective degradation of some centrosome-associated proteins that inhibit the initiation of ciliogenesis.

**Figure 4:**
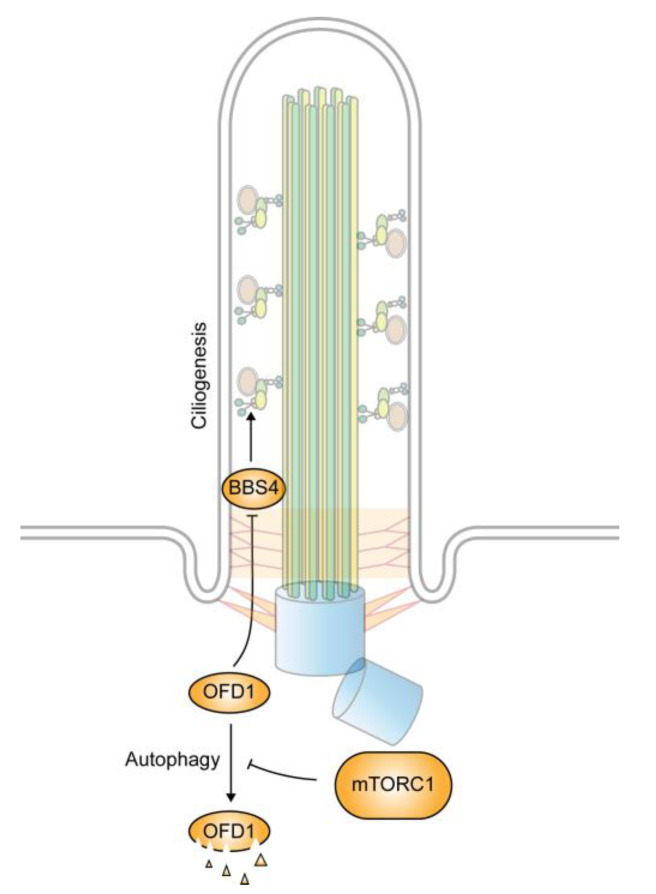
Regulation of ciliogenesis by autophagy. Increased autophagy under starvation condition promotes ciliogenesis by the selective degradation of oral–facial–digital syndrome type 1 (OFD1), an inhibitor of BBSome formation. The autophagy-mediated degradation is negatively regulated by mTORC1.

The oral–facial–digital syndrome type 1 (OFD1) protein is a centrosome/basal body associated protein localized at both the centriole and centriolar satellites [97,98]. Mutations in the OFD1 gene cause OFD syndrome [99]. During ciliogenesis, OFD1 functions to promote the docking of the basal body to the plasma membrane and is involved in the recruitment of IFT88, an essential component of the IFT particles, to the basal body [100]. In cells subjected to serum starvation, the activation of autophagy decreases the level of OFD1 at the centriolar satellite, but not that at centrioles. The specific reduction in OFD1 allows for the presentation of BBS4 in cilia. When autophagy is blocked, OFD1 accumulates at the centriolar satellites and impedes the recruitment of BBS4, leading to a blockage of ciliation [98] (Figure 4). The knockdown of OFD1 is able to restore the ciliation defect in the autophagy-deficient cells. In addition, OFD1 depletion can also lead to ciliation in cycling and transformed MCF-7 human breast cancer cells that do not normally express cilia. These observations suggest that the autophagy-mediated degradation of OFD1 is a key step in ciliogenesis [98]. Consistent with the view, it has been found that the upregulation of autophagy activity elongates cilia, whereas the downregulation of autophagy shortens them [101]. However, under normal nutrient conditions, a basal level of autophagy appears to have a negative role in ciliogenesis. It does so by selective degradation of IFT20 [102]. IFT20 is an essential component of the IFT-B complex that is involved in vesicle transport from the Golgi apparatus to cilia [103]. By keeping its level low, autophagy hence prevents ciliogenesis.

The selective degradation of key components involved in ciliogenesis by autophagy represents a unique mechanism that couples growth factor and nutrient conditions with ciliogenesis. As a key regulator of autophagy, it is anticipated that mTORC1 is able to act through an autophagy-dependent mechanism to control ciliogenesis. However, it remains unclear to what extent mTORC1 relies on this autophagy-dependent mechanism to control ciliogenesis.

## 8. Cilium-Dependent Regulation of Autophagy

While autophagy is able to control ciliogenesis, defects in primary cilia also have a profound impact on autophagy activity. The link between primary cilia and autophagy is conceivable since autophagy is responsive to mTORC1 activity, which is controlled by primary cilia. In normal ciliated cells, flow stress increases autophagy activity and reduces cell size. The effect is mediated by cilia through the LKB1–AMPK signaling axis, which leads to the downregulation of mTORC1 [104]. Other mTORC1-dependent mechanisms also exist. The depletion of Retinitis pigmentosa GTPase regulator-interacting protein-1-like (RPGRIP1L), which is localized at the transition zone of primary cilia, reduces autophagy by activating mTORC1, presumably through Akt [105].

In addition to controlling autophagy indirectly through mTORC1-dependent mechanisms, primary cilia also have a direct role in the regulation of autophagy. Many autophagy-related proteins (ATGs) are found to localize within the ciliary compartment, including Atg16L, AMBRA-1, LC3, GABARAP, and Vps15. Several other ATGs, including Atg5, Vps34, Atg7, and Atg14 are also found to associate with the basal body. When ciliogenesis is blocked, this ciliary distribution of autophagic machinery is disrupted, which results in reduced autophagosome formation upon serum starvation [102]. However, defects in cilia do not appear to affect the basal autophagic flux under normal growth conditions, suggesting that primary cilia are required only for inducible autophagy.

The autophagy defect in cilium deficient cells cannot be rescued by rapamycin treatment, indicating that it is not caused by the abnormal activation of mTORC1. However, increasing Shh signaling in the cilium-deficient cells is able to restore autophagy under the starvation condition [102]. This observation suggests that Shh signaling plays a positive role in autophagy. How activated Shh signaling promotes autophagy remains unknown. It has been shown that Shh signaling stimulates autophagy by increasing the expression of autophagy-related genes [106]. In this regard, the effect of Shh signaling on autophagy in the cilium-deficient cells is likely to be mediated indirectly through Shh-signaling-mediated transcription.

## 9. Conclusions

Evidence accumulated so far suggests that primary cilia play a negative role in mTORC1 signaling by converting mechanical stimuli into inhibitory activities. Multiple mechanisms have been identified for the conversion. However, the LKB1/AMPK dependent mechanism is the only one with evidence showing a prompt response of mTORC1 to flow stress. The others are built largely on a correlation between PC-1 dysfunctions and an abnormal mTORC1 activation. The LKB1/AMPK mechanism operates through the flow stress-induced ciliary accumulation of LKB1, which subsequently activates AMPK localized at the basal body. However, it remains unknown how mechanic signals arising from the bending of cilia activate LKB1. The most recent evidence suggests that mechanical signals that activate LKB1 may be mediated by PC-1. However, how PC-1 transmits the signal to LKB1 is yet to be determined.

In addition to the mechanosensory function, primary cilia also harbor PDEGF receptors and many GPCRs. These receptor-mediated signaling pathways are likely to participate in the cilium-dependent regulation of mTORC1 and may crosstalk with the mechano-responsive mechanisms of cilia. However, as these receptors also exist outside of primary cilia, the challenge lies in separating cilium-dependent effects from those that are independent.

While changes in mTORC1 activity affect ciliogenesis and stability, there is no evidence for a direct role of mTORC1 in the processes. Current evidence suggests that the effect of mTORC1 on ciliation is mediated indirectly through its role in autophagy. The mTORC1-responsive autophagy activity controls ciliogenesis through the selective degradation of inhibitory components involved in the process. In this way, autophagy functions as a positive regulator of ciliogenesis and different levels of autophagy can regulate cilium assembly and maintenance by targeting the turnover of various ciliary proteins.
